# Incidence of Unplanned Readmissions After One-Day Surgery Discharge Among Pediatric Patients: A Retrospective Study From a Tertiary Care Hospital in Saudi Arabia

**DOI:** 10.7759/cureus.59896

**Published:** 2024-05-08

**Authors:** Yasser M Almashari, Yazeed Allarakia, Omar Barradah, Yazan Alqozi, Abdullah M Almoraei, Rand A Alshaya, Rayan Muawad

**Affiliations:** 1 College of Medicine, King Saud Bin Abdulaziz University for Health Sciences, Riyadh, SAU; 2 Pediatric Anesthesia, Ministry of National Guard - Health Affairs, Riyadh, SAU

**Keywords:** outpatient surgery, same day discharge, unplanned readmission, anesthesia, complication, day surgery

## Abstract

Introduction

The prevalence of one-day surgery (also known as same-day surgery or outpatient surgery) has been increasing recently among patients and physicians in many countries due to its benefits. The main benefits of one-day surgery are that the patient is not planned to stay overnight before the surgery and can be discharged on the same day of the surgery. The lower cost to the health system can make these surgeries more favorable for both sides. However, unplanned readmission after such surgeries can happen and this has broad implications for patients, their families, and the healthcare system. Therefore, this study primarily aims to identify the incidence of unexpected hospital readmissions following one-day surgery after discharge among children. The study also aims to identify any significant variables that can be identified with the cases of readmissions to allow for further investigations in future studies

Methods

This study was done at King Abdullah Specialist Children’s Hospital in Riyadh, Saudi Arabia. The target population included all pediatric patients who underwent one-day surgeries and were admitted within one week of their discharge from 2017 to 2023 through outpatient clinics and the emergency department.

Results

The study sample size was 403 patients, with male patients accounting for 241 surgeries (59.8%), and female patients accounting for 162 surgeries (40.1%). The most common American Society of Anesthesiologists (ASA) classification was II, accounting for 169 cases (41.9%). Toddlers and preschoolers (aged 1-6 years) were the age groups with the highest number of patients (n=252, 62.5% combined). Elective surgeries accounted for 382 cases (94.7%). The specialty with the highest number of surgeries was ear, nose, and throat with 284 cases (70.4%) with tonsillectomy with adenoidectomy being the most common surgery with 234 cases (58%). The most common reasons for unplanned readmission were poor oral intake (n=146, 36.2%) and bleeding (n=131, 32.5%). The most common day of readmission was the seventh day in five surgical specialties (45.4%).

Conclusion

Over the past seven years, 403 patients were readmitted within one week after their one-day surgery at King Abdullah Specialist Children’s Hospital. Such a situation may cause dissatisfaction with the medical care that the patients were given and eventually may build an untrusted relationship between the patient and the physician. Future investigations should be established to lower such a condition and develop prevention methods to lower its prevalence.

## Introduction

One-day surgery (also known as same-day surgery or outpatient surgery) has been increasing in many countries due to its benefits which include no hospital stay and lower cost to the health system. However, there can be unplanned readmissions after such surgeries, which affect not only the patients but their families and the healthcare system. Moreover, the rate of unplanned postoperative readmission can serve as a meaningful indicator of the quality of care provided by a medical facility [1-3]. Unplanned readmissions can be attributed to various factors, including surgical complications, anesthesia complications, and social factors [1-5]. For example, recent studies showed that the reasons for unplanned readmissions were surgical complications like bleeding [4]. Other studies showed that anesthetic complications like PONV also rose to be the primary cause of readmission in multiple cases and late surgery as a social cause [5].

Surgical specialties also must be considered. For example, in a study with 4272 cases, 11 readmissions were identified in General Surgery and Otolaryngology accounting for 91% of the readmissions, and plastic surgery accounted for 9% [6]. The type of procedure can also give a hint about the probable cause of readmission. In a study that was conducted on 3,669 pediatric patients, 128 were admitted following endoscopic sinus surgery, and 17.2% of the patients were admitted due to epistaxis [7]. In another study on pediatric tonsillectomy patients, 77.5% revisited the emergency department and 16.2% were readmitted as inpatients due to bleeding [8]. It is important to identify patient- and surgery-related factors for readmission following one-day surgeries to optimize patient care, enhance healthcare quality, allow for efficient resource allocation, reduce economic burden, and improve patients’ well-being. Therefore, this study primarily aims to identify the incidence of unexpected hospital readmissions following one-day surgery discharge among children. The study also aims to identify any significant variables that can be identified with readmission cases to allow for further investigations in future studies.

## Materials and methods

This was a retrospective cross-sectional study conducted at King Abdullah Specialist Children’s Hospital in Riyadh, Saudi Arabia. The study was approved by King Abdullah International Medical Research Center (approval number: IRB/1197/23). The target population included all pediatric patients who underwent one-day surgery at King Abdullah Specialist Children’s Hospital and were readmitted within a week of their discharge between 2017 and 2023. We excluded patients who were admitted after the first week to ensure that the admission was related to the procedure. Patients who were not admitted through the emergency department or outpatient clinics were also excluded.

Data were collected from the hospital software that automatically stores patient data, ensuring the integrity of data collection. The data were subsequently entered into a Microsoft Excel spreadsheet (Microsoft Corporation, Redmond, Washington, United States). Python libraries including SciPy, Metplotlib, Seaborn, and pandas were used for data analysis, with variables including age, sex, surgery type, anesthesia type, case type, the day the patient was readmitted after discharge, cause of admission, American Society of Anesthesiologists (ASA) score, and the specialty of the surgeon who conducted the surgery. Based on the results of the data analysis, we aimed to obtain insights into the prevalence of readmission following one-day surgery in terms of computed frequencies, percentages, means, medians, and standard deviations (SD) of the study variables. Density plots were generated to visualize the distribution of variables, including means and ranges (i.e., one SD from the mean), to illustrate the variability and main trends of the data.

## Results

A total of 403 patients were included in the study. As shown in Table 1, the study sample was predominantly male (n=241, 59.8%). The most common ASA physical status classification was II, accounting for 41.9% of cases. The toddler (aged 1-3 years) and preschooler groups (aged 1-6 years) had the same number of cases, each accounting for 31.26% (n=126) of patients, making them the most common age groups.

**Table 1:** Patient demographics

Characteristics		Count (n)	Percentage (%)
Sex	Male	241	59.8
	Female	162	40.1
American Society of Anesthesiologists classification	I	165	40.9
	II	169	41.9
	III	39	9.6
	Ie	15	3.7
	IIe	10	2.4
	IIIe	3	0.7
	IV	2	0.4
Age	Newborn (0–1 month)	2	0.4
	Infant (1 month to 1 year)	18	4.4
	Toddlers (1–3 years)	126	31.2
	Preschoolers (3–6 years)	126	31.2
	School-age children (6–12 years)	101	25.0
	Adolescents (12–19 years)	30	7.4

**Figure 1:**
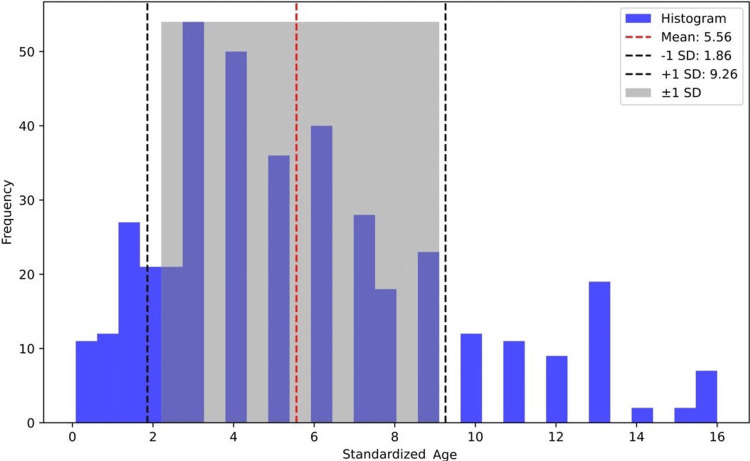
Age distribution for surgical patient readmissions The mean (dashed red line) indicates the average age of readmitted patients (i.e., approximately 5.56 years), anchoring the distribution. The density (solid blue line) represents the distribution of patient ages at readmission. The curve’s shape and peak positions suggest common ages for readmission. The section marked ±1 SD (shaded area) covers ages of approximately 22 months to 111 months (i.e., 1.86-9.25 years), indicating the most densely populated age range for readmissions.

The mean age of the sample was 5.56 years, with an SD of 3.7 (approximately 1.86-9.25 years), as shown in Figure 1.

The majority of surgeries (n=382, 94.7%) were classified as elective. The specialty with the highest number of surgeries was ear, nose, and throat (ENT), with 284 cases (70.4%), underscoring the importance of ENT procedures within the surgical landscape of the hospital. All cases received general anesthesia during surgery. Table 2 provides more details about case type, anesthesia type, and surgery specialty.

**Table 2:** Case type, surgery specialty, and anesthesia type ENT: ear, nose, and throat

		Count (n)	Percentage (%)
Case type	Elective	382	94.7
	Emergency	21	5.2
Anesthesia type	General	403	100
Subspecialty	ENT	284	70.4
	Orthopedic	37	9.1
	Urology	27	6.6
	Pediatric surgery	25	6.2
	Dentistry	8	1.9
	Neurosurgery	6	1.4
	Ophthalmology	6	1.4
	Plastic surgery	3	0.7
	Gastroenterology	3	0.7
	General surgery	3	0.7
	Cardiac surgery	1	0.2

Table 3 lists the causes of unplanned readmission, which were predominantly poor oral intake with 146 cases (36.2%) and bleeding with 131 cases (32.5%). Planned admissions accounted for 51 cases (21.6%). Anesthesia complications also were associated with readmission, with 11 cases (2.7%).

**Table 3:** Causes of unplanned readmission PONV: postoperative nausea and vomiting; UTI: urinary tract infection; CSF: cerebrospinal fluid

	Count (n)	Percentage (%)
Poor oral intake	146	36.2
Bleeding	131	32.5
Continuity of care (planned)	51	12.6
Pain	18	4.4
PONV	11	2.7
Fever	9	2.2
Surgical site infection	8	1.9
Vomiting	6	1.4
Surgical site swelling	6	1.4
Upper respiratory symptoms (e.g., sneezing, cough, runny nose)	5	1.2
Medical condition complication	4	0.9
UTI	3	0.7
Upper respiratory tract infection	2	0.4
CSF leak	2	0.4
Acute gastroenteritis	1	0.2

Table 4 shows the causes of unplanned readmission according to specialty. The most common cause of readmission after dentistry and ENT surgeries was poor oral intake, whereas those of neurosurgeries and general surgeries were cerebrospinal fluid (CSF) leak and pain, respectively. Planned readmission (continuity of care) was the most common cause of readmission in cases of ophthalmology (50%), urology (22.2%), orthopedic (67.7%), gastroenterology (100%), and pediatric (32%) surgeries.

**Table 4:** Causes of unplanned readmission according to specialty PONV: postoperative nausea and vomiting; CSF: cerebrospinal fluid; UTI: urinary tract infection

Specialty	Cause	Count (n)	Percentage (%)
Dentistry	Poor oral intake	2	25.0
	Continuity of care	2	25.0
	Fever	1	12.5
	Upper respiratory symptoms	1	12.5
	PONV	1	12.5
	Bleeding	1	12.5
Ear, nose, and throat	Poor oral intake	135	47.5
	Bleeding	127	44.7
	Pain	8	2.8
	Continuity of care	3	1.0
	PONV	3	1.0
	Upper respiratory tract infection	2	0.7
	Vomiting	2	0.7
	Surgical site infection	2	0.7
	Upper respiratory symptoms	1	0.3
	Fever	1	0.3
Neurosurgery	CSF leak	2	33.3
	PONV	1	16.6
	Surgical site swelling	1	16.6
	Vomiting	1	16.6
	Medical condition complication	1	16.6
Ophthalmology	Continuity of care	3	50.0
	Upper respiratory symptoms	1	16.6
	PONV	1	16.6
	Fever	1	16.6
Orthopedic	Continuity of care	25	67.5
	Poor oral intake	4	10.8
	PONV	2	5.4
	Fever	1	2.7
	Pain	1	2.7
	Bleeding	1	2.7
	Vomiting	1	2.7
	Surgical site infection	1	2.7
	Upper respiratory symptoms	1	2.7
Urology	Continuity of care	6	22.2
	Fever	4	14.8
	Surgical site swelling	4	14.8
	UTI	3	11.1
	Surgical site infection	3	11.1
	PONV	2	7.4
	Poor oral intake	2	7.4
	Medical condition complication	1	3.7
	Pain	1	3.7
	Bleeding	1	3.7
Cardiac surgery	Fever	1	100
Gastroenterology	Continuity of care	3	100
General surgery	Pain	2	66.6
	Poor oral intake	1	33.3
Pediatric surgery	Continuity of care	8	32.0
	Pain	5	20.0
	Medical condition complication	2	8.0
	Poor oral intake	2	8.0
	Vomiting	2	8.0
	Bleeding	1	4.0
	Acute gastroenteritis	1	4.0
	Upper respiratory symptoms	1	4.0
	Surgical site swelling	1	4.0
	PONV	1	4.0
	Surgical site infection	1	4.0
Plastic surgery	Surgical site infection	1	33.3
	Continuity of care	1	33.3
	Pain	1	33.3

ENT surgeries such as tonsillectomy with or without adenoidectomy, and adenoidectomy without tonsillectomy, emerged as the surgical interventions with the highest rate of readmission, which likely reflects their prevalence as commonly indicated procedures within the clinical context of the hospital, as seen in Table 5.

**Table 5:** Surgeries with the highest rates of readmission DDH: developmental dysplasia of the hip; EDH: epidural hematoma; DHL: distal hamstring lengthening; PCN: percutaneous nephrostomy

Surgery name	Count (n)	Percentage (%)
Tonsillectomy with adenoidectomy	234	58.0
Tonsillectomy without adenoidectomy	22	5.4
Adenoidectomy without tonsillectomy	14	3.4
Open reduction for developmental dislocation of hip DDH	11	2.7
Full dental clearance	8	1.9
Cystoscopy	5	1.2
Repair of inguinal hernia, unilateral	5	1.2
Pelvic osteotomy and open reduction for DDH	5	1.2
Posteromedial release of clubfoot	4	0.9
Myringotomy with insertion of tube, bilateral	4	0.9
Esophagoscopy	4	0.9
Male circumcision	4	0.9
Control of hemorrhage	4	0.9
Orchidopexy for undescended testis, unilateral	3	0.7
Orchidopexy for undescended testis, bilateral	3	0.7
Laparoscopic appendicectomy	3	0.7
Repair of umbilical hernia	2	0.4
Lengthening of Achilles’ tendon	2	0.4
Reconstruction of congenital vertical talus	2	0.4
Insertion of ventriculoperitoneal shunt	2	0.4
Pelvic examination under anesthesia	2	0.4
Implantation of cochlear prosthetic device	2	0.4
Hypospadias, staged repair, first stage	2	0.4
Urethral meatotomy	2	0.4
Central vein catheterization	2	0.4
Ureteroscopic lithotripsy	2	0.4
Revision of ventricular shunt	2	0.4
Retinal photography of both eyes	1	0.2
Closed reduction of fracture of shaft of radius	1	0.2
Plantar fasciotomy	1	0.2
Closed reduction of dislocation of hip	1	0.2
Primary closure of laceration	1	0.2
Probing of lacrimal passages, bilateral	1	0.2
Pyeloplasty	1	0.2
Radical excision of lymph nodes of neck	1	0.2
Radical nephrectomy	1	0.2
Recession of one extraocular muscle	1	0.2
Change of hip spica cast to broomstick cast	1	0.2
Release of contracture of fingers	1	0.2
Removal of plate from femur	1	0.2
Strabismus procedure involving three or more muscles, one eye	1	0.2
Strabismus procedure involving one or two muscles, both eyes	1	0.2
Sigmoidectomy	1	0.2
Closed reduction for developmental dislocation of hip DDH	1	0.2
Rigid bronchoscopy	1	0.2
Repair of myelomeningocele	1	0.2
Repair of inguinal hernia, bilateral	1	0.2
Craniotomy and EDH removal	1	0.2
Osteotomy of proximal femur with internal fixation	1	0.2
Incision and drainage, abscess, perianal	1	0.2
Definitive intestinal resection and pull-through anastomosis	1	0.2
Dilation of esophagus by endoscopy	1	0.2
Distal hamstring lengthening DHL	1	0.2
Distal hypospadias, single stage repair	1	0.2
Endoscopic balloon dilation of esophagus	1	0.2
Endoscopic resection of a single lesion of bladder ≤2 cm	1	0.2
Corrective osteotomy	1	0.2
Esophagoscopy and gastroscopy	1	0.2
Excision of submandibular gland	1	0.2
Excisional debridement of soft tissue	1	0.2
Hypospadias, staged repair, second stage	1	0.2
Incisional biopsy of soft tissue mass	1	0.2
Orchidectomy, unilateral	1	0.2
Laparoscopic cholecystectomy	1	0.2
Laparoscopic excision of lesion of pelvic cavity	1	0.2
Laparoscopy	1	0.2
Laryngoscopy	1	0.2
Amputation of finger	1	0.2
Manipulation/mobilization of joint	1	0.2
Colonoscopy	1	0.2
Nephrolithotomy, percutaneous using established PCN	1	0.2
Closure of ventricular septal defect	1	0.2
Open reduction of fracture of femur with internal fixation	1	0.2
Corrective wedge osteotomy, cuboid	1	0.2
Ophthalmological examination under anesthesia	1	0.2

**Figure 2:**
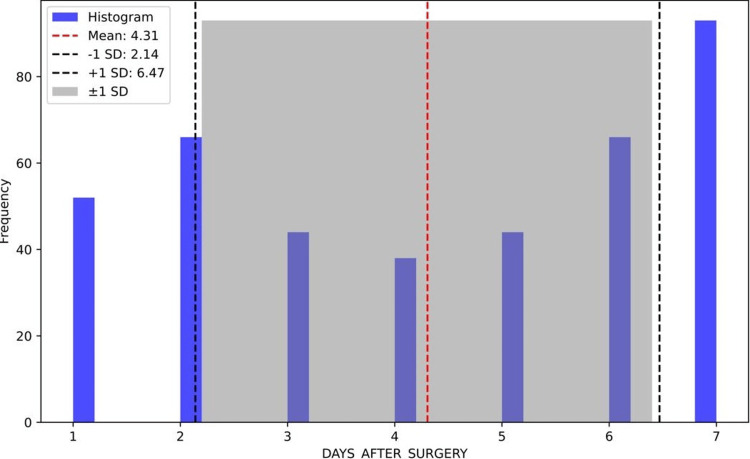
Distribution of readmission days post surgery The mean (dashed red line) marks the average number of days to readmission after surgery, at approximately 4.31 days. This value indicates a central tendency around which readmissions are most densely clustered. The density (solid blue line) portrays the probability density of readmissions on different postoperative days, where higher peaks indicate a higher rate of readmission are around that day. ±1 SD (Shaded Area): Covers the span from around 2.14 to 6.47 days post surgery, encompassing the range within which the bulk (approximately 68%) of readmission days fall.

Notably, our investigation revealed that readmissions occurred at the highest frequency on postoperative day 7. The mean number of days to readmission after surgery was approximately 4.30 days, with a standard deviation of 2.16, and 68% of cases were readmitted at approximately 2.14-6.47 days postoperatively, as seen in Figure 2.

For surgeries in the specialties of ENT, neurosurgery, orthopedics, cardiology, and pediatric surgery, the most common postoperative day for readmission was the seventh day, whereas that of dentistry and ophthalmology surgeries was the first day, as shown in Table 6.

**Table 6:** Postoperative day of readmission according to specialty

Specialty	Day of readmission after discharge	Count (n)	Percentage (%)
Dentistry	1	3	37.5
	6	3	37.5
	2	1	12.5
	7	1	12.5
Ear, nose, and throat	7	57	20.0
	2	54	19.0
	6	48	16.9
	1	37	13.0
	3	31	10.9
	4	29	10.2
	5	28	9.8
Neurosurgery	7	4	66.6
	2	1	16.6
	5	1	16.6
Ophthalmology	1	2	33.3
	7	1	16.6
	2	1	16.6
	3	1	16.6
	5	1	16.6
Orthopedics	7	18	48.6
	5	5	13.5
	3	4	10.8
	6	4	10.8
	2	3	8.1
	1	2	5.4
	4	1	2.7
Urology	6	6	22.2
	5	6	22.2
	1	4	14.8
	7	3	11.1
	4	3	11.1
	3	3	11.1
	2	2	7.4
Cardiology	7	1	100
Gastroenterology	6	2	66.6
	5	1	33.3
General surgery	3	1	33.3
	2	1	33.3
	7	1	33.3
Pediatric surgery	7	7	28.0
	4	4	16.0
	3	4	16.0
	1	3	12.0
	2	3	12.0
	6	2	8.0
	5	2	8.0
Plastic surgery	6	1	33.3
	4	1	33.3
	1	1	33.3

## Discussion

The main goal of this study was to identify the prevalence of unplanned admissions which means any admissions for the same or a related diagnosis after discharge in King Abdullah Specialist Children’s Hospital. A total of 403 postoperative readmissions met the inclusion criteria and were included in the study. The average readmitted patient age was approximately five years, with a standard deviation of 3.70 years (Figure 1). As seen in Table 1, readmission was more prevalent in male patients (n=241, 59.8%) compared to female patients (n=162, 40.19%), which agrees with findings reported by Alghamdi et al. [1] and Broch et al. [2]. The age of the study participants ranged from one month to 16 years. The highest rate of readmission was seen in the younger pediatric group, especially toddlers and preschoolers (n=252, 62.4%) combined, These results contraindicated with those of Murto et al. who found that the mean age was seven years [3].

The ASA classification scores in our study were mostly grade I and II accounting for 169 and 165 readmissions, respectively, together representing approximately 80% of cases, which correspond with the findings of de Souza Neto et al. [6] and Alghamdi et al. [1]. Furthermore, the high number of low ASA score cases among our patients is due to most of our patients in one-day surgery being categorized in these two scores, and patients with high ASA scores are less commonly seen in one-day surgeries. 

After categorizing our readmission cases according to the surgical specialties of the one-day surgeries, we found that ENT had the highest readmission rate (n=284, 70.4%). On the other hand, the specialty with the least readmissions was cardiac surgery with only one (0.25%) case. This correlates with Awad et al.'s study which established that ENT was the specialty with the highest number of readmissions, with 21 cases out of 2026 ENT surgeries [5]. The high readmission rate of ENT surgeries is attributed to tonsillectomies, either with or without adenoidectomy. Shay et al. reported a similar finding, with 16.2% readmitted out of 36,221 cases in their study [8]. Moreover, the high number of readmissions under this type of surgery is also due to the fact that it is a more common surgery than the rest.

According to our analysis, the main causes of unplanned readmission were poor oral intake (n=146, 36.2%), bleeding (n=131, 32.5%), and pain (n=18, 4.4%). These results agreed with those of Awad et al. [5] and Shay et al. [8], who also reported postoperative bleeding and pain as leading causes of readmission [5,8]. Continuity of care was the only cause found for planned readmissions in this study, with a patient count of 51 (12.65%), similar to the study by Awad et al. [5].

The variety of causes of the unplanned readmissions were due to the the different types of specialties and surgeries that we included in our study. If we look at the results of each specialty we can see each one of them has almost the same causes and even the same days of readmissions, and since we have such a clear similarity between them, a systematic approach to prevent such a condition should be established to all the surgical specialties. Identifying what exactly went wrong where, or what can be improved between the time of admission to the post-anesthesia care unit (PACU) and the discharge should be a priority to to avoid unplanned readmission and raise the level of satisfaction of medical care provided to patients.

Limitations

As much as we tried to make our study flawless, there are still several limitations. Firstly, the sample population was taken from only one region (Riyadh, Saudi Arabia), which limits the representativeness of our results. Secondly, no control group was added. Thirdly, due to the high number of surgeries and surgery types in our study, each case may not necessarily be written the exact same way as the others in the hospital software, which may lead to confusion regarding whether some surgeries are related to the study criteria or not, hence a prediction chart could not be made. Chronic diseases were not considered in this study, which could have been useful to further explore the reasons for unplanned readmissions. Finally, we could not confirm whether the type of anesthesia had any correlation with unplanned readmissions, as only one type of anesthesia was used in the cases of our study. 

## Conclusions

Over the past seven years, 403 patients were readmitted within one week after their one-day surgery at King Abdullah Specialist Children’s Hospital. Such a situation may cause dissatisfaction with the medical care that the patients were given and eventually may build mistrust between the patient and the physician. Future investigations should be established to lower such a condition and develop prevention methods to lower its prevalence. Investigations should include specialties and surgeries with a high number of readmissions and identify the causes. An onsite or virtual follow-up with the patient needs to be carried out that emphasizes how important it can be to the family to identify the causes early and receive the necessary care at an early stage to prevent readmission.

## References

[1] Alghamdi AM, Aljadaan SA, Alsemairi SA, Alowairdhi MA, Alhussain MA, Alrumyyan RA (2022). Safety and readmission in pediatric ambulatory surgery in a tertiary hospital. Cureus.

[2] Broch A, Paye-Jaouen A, Bruneau B (2023). Day surgery in children undergoing retroperitoneal robot-assisted laparoscopic pyeloplasty: is it safe and feasible?. Eur Urol Open Sci.

[3] Murto KT, Katz SL, McIsaac DI, Bromwich MA, Vaillancourt R, van Walraven C (2017). Pediatric tonsillectomy is a resource-intensive procedure: a study of Canadian health administrative data. Can J Anaesth.

[4] Van Caelenberg E, Benoit D, Verhaeghe R, Coppens M (2022). Unanticipated admission after ambulatory surgery in the pediatric population: a single-center retrospective analysis. Acta Chir Belg.

[5] Awad IT, Moore M, Rushe C, Elburki A, O'Brien K, Warde D (2004). Unplanned hospital admission in children undergoing day-case surgery. Eur J Anaesthesiol.

[6] de Souza Neto EP, Martinez JL, Dekoven K, Ruest P, Girard MA (2019). Predictors of unanticipated admission in paediatric patients after ambulatory surgery. EC Anaesth.

[7] McKeon M, Medina G, Kawai K, Cunningham M, Adil E (2019). Readmissions following ambulatory pediatric endoscopic sinus surgery. Laryngoscope.

[8] Shay S, Shapiro NL, Bhattacharyya N (2015). Revisit rates and diagnoses following pediatric tonsillectomy in a large multistate population. Laryngoscope.

